# The armoured cuticle of the black soldier fly *Hermetia illucens*

**DOI:** 10.1038/s41598-023-49549-5

**Published:** 2023-12-13

**Authors:** Manuela Rebora, Gianandrea Salerno, Silvana Piersanti, Valerio Saitta, Diletta Morelli Venturi, Chuchu Li, Stanislav Gorb

**Affiliations:** 1 Dipartimento di Chimica, Biologia e Biotecnologie, University of Perugia, Via Elce di Sotto 8, 06121 Perugia, Italy; 2 Dipartimento di Scienze Agrarie, Alimentari e Ambientali, University of Perugia, Borgo XX Giugno, 06121 Perugia, Italy; 3 Department of Functional Morphology and Biomechanics, Zoological Institute, Kiel University, Am Botanischen Garten 9, 24098 Kiel, Germany

**Keywords:** Zoology, Materials science

## Abstract

We characterise in detail the larval and pupal cuticle of the black soldier fly *Hermetia illucens* L. (Diptera: Stratiomyidae)*,* a key insect species in circular economy. In particular, we focus on ultrastructure using scanning and transmission electron microscopy, material characterization and composition (elements and minerals) with confocal laser scanning microscope, energy dispersive X-ray microanalysis, powder X-ray diffraction and mechanical properties with nanoindentation measurements. Calcium carbonate crystallizes on the epicuticle as blocks of calcite in the pupal cuticle. Calcium carbonate granules are stored in two specialised Malpighian tubules. CaCO_3_ is already present in the cuticle of young larval instars, but it is mainly in the form of amorphous calcium carbonate while the amount of calcite increases during larval development. The presence of calcite leads to cuticle hardening. Larval and pupal cuticles contain large amounts of resilin which guarantee cuticle flexibility.

## Introduction

Biomineralization and deposition of calcium salts in arthropod cuticle are typical features of Crustacea like lobsters and crabs, whose cuticle is constituted of chitin-protein organic fibres associated with calcite and amorphous calcium carbonate (ACC) and amorphous calcium phosphate (ACP) (review in Ref.^1^). Calcium concentration is very high in seawater and these animals can use it to make their integument harder against mechanical stress and serving as armour against predators. In terrestrial environments, where the availability of calcium at ecdysis can be high, but also low or absent, terrestrial crustaceans, such as species of the genus *Porcellio* and other isopods, have developed different strategies to keep the calcium, such as storing calcium during the pre-moult period as ACC and feeding on the old own cuticle^2–5^. Among insects, the great conquerors of emerged land, cuticle biomineralization is surely less common than in crustaceans. The cuticle of the mandible of the leaf-cutting ant *Atta sexdens rubipilosa*^6^ and stored product beetles^7^ or the wood penetrating ovipositor of parasitoids^8^ and cicadas^9^ can be impregnated with heavy metal ions, such as Zn, Mn, or occasionally Fe, present in relatively large amounts and increasing cuticle hardness significantly^10^. Overall, it is possible to assess that, in insects, heavy metal ions to strengthen cuticle are preferred to mineralisation with Ca-salts^11^. While cuticle calcification is nearly ubiquitous in non-hexapod Pancrustacea, it is extremely rare in insects^1^ where a lighter cuticle, compared with that of Crustacea, developed for adaptation to terrestrial and aerial environments^12^. Some exceptions to this can be envisaged among Diptera. The major components (62%) of the puparium of *Musca autumnalis* De Geer (Diptera: Muscidae) are calcium, magnesium, phosphate and carbonate^13^. The pupal cuticle of *Bactrocera dorsalis* (Hendel) (Diptera: Tephritidae) is stiffened by ACC with a high level of magnesium, mainly distributed in the exocuticle^14^. In the exoskeleton of the xylophagid fly larva *Exeretonevra angustifrons* Hardy (Diptera: Xylophagidae), the epicuticle of the body shows minute hemispherical protrusions of amorphous calcium phosphate in the epicuticle^15^. The larvae of different subfamilies of Stratiomyidae and Xylomyidae, both belonging to the infraorder Stratiomomorpha, show mineralised scales on their body. This feature is a synapomorphy of these two families^16,17^. The presence of calcium carbonate in the integument of stratiomyiid larvae and their puparia has been described in very old papers dating to the end of the XIX century and the beginning of the XX century (see review in Ref.^18^). It is reported^19^ that the calcium carbonate in stratiomyid larvae (*Sargus cuprarius* L. (Diptera: Stratiomyidae)) is deposited in the integument after each moult, as “calcareous warts embedded in shallow pits” originated in the Malpighian tubules (MTs). Different drawings are reported on this old description but, since then, to the best of our knowledge, no detailed investigation was performed on the armoured cuticle of these insects, in spite of the large number of articles published in the last years on *Hermetia illucens* L. (Diptera: Stratiomyidae), the ‘crown jewel’ of the insects as feed industry species^20^.

*H. illucens*, or black soldier fly (BSF), is a common cosmopolitan species whose larvae are decomposers able to break down a huge amount of organic matter (e.g. fresh manure and food wastes of both animal and vegetable origin) and generate valuable biomass^21^. For its important role in the circular economy, this insect species is gaining interest worldwide (see review in Ref.^22^). Flours obtained from BSF larvae, thanks to their high content of fat, protein and high-quality amino acids and minerals^23,24^, can be used as a feed source, ultimately helping to solve the global food problem. The materials that can be extracted from BSF larvae have potential applicability also in cosmetics^25^ and extracted crude fat from these insects can be converted into biodiesel^26^.

In this context, the aim of the present investigation is to describe in detail the cuticle of BSF larva at different developmental instars with different techniques. The data here reported concern in particular: ultrastructure using scanning and transmission electron microscopy (SEM, TEM), material composition and chemical characterisation (elements and minerals) with confocal laser scanning microscope (CLSM), energy dispersive X-ray microanalysis (EDX), powder X-ray diffraction (PXRD) and mechanical properties (hardness and elasticity) with nanoindentation measurements. A detailed investigation on the structure and material composition of the cuticle of BSF larva can help to widen the basic knowledge on biomineralization in insects and add important information on the use of BSF larvae as mineral source in animal diet.

## Results

**Figure 1:**
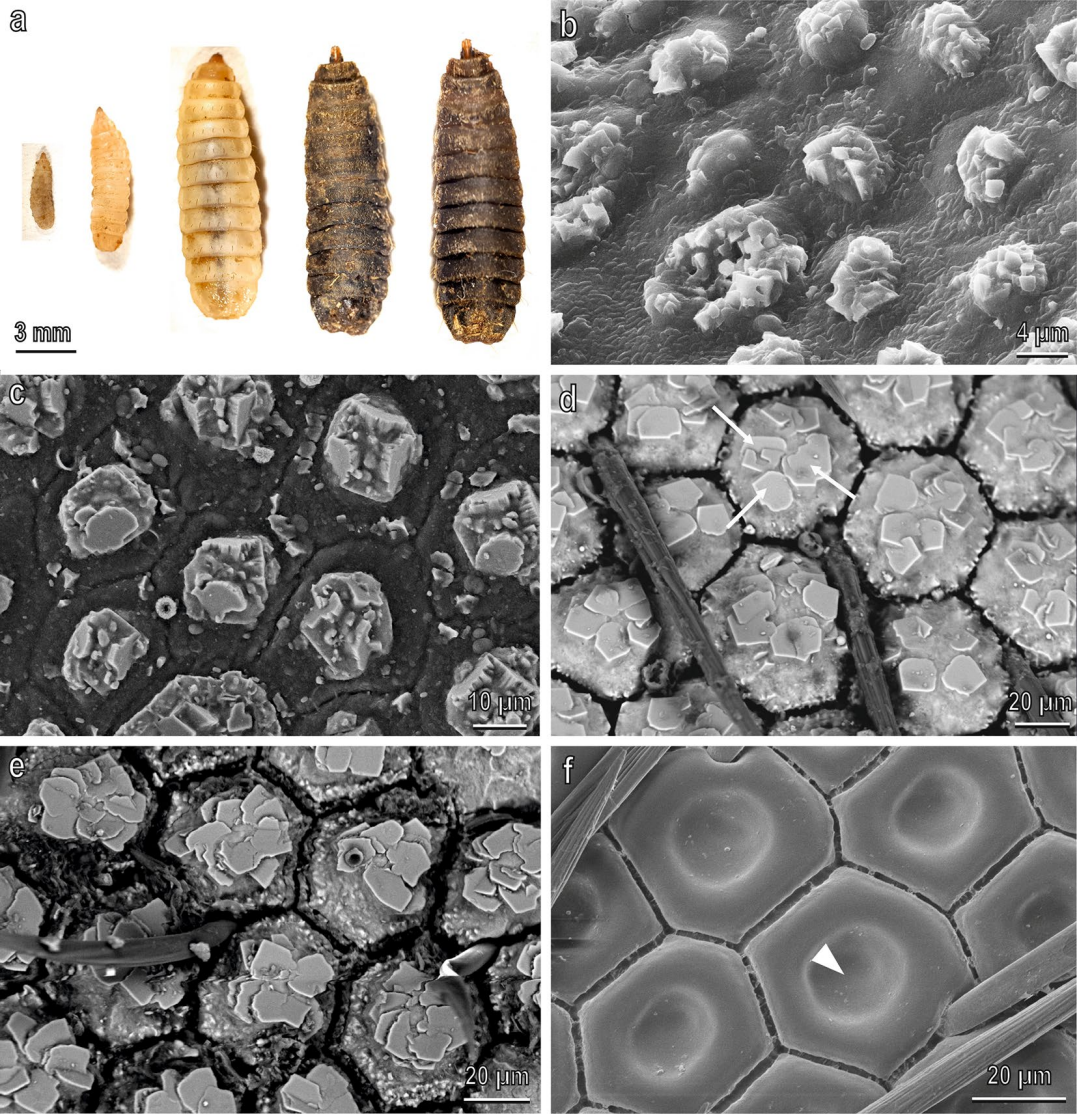
*H. illucens* larval instars in light microscope (**a**) and in SEM ((**b**–**e**), back scattered electrons, brighter areas indicate presence of CaCO_3_ owing to its higher atomic number composition material compared to cuticle; (**f**), secondary electrons). (**a**) from the left to the right: larva with a size of 2–3 mm, larva with a size of 5 mm, clear larva with a size of 10–15 mm, dark larva with a size of 10–15 mm (prepupa); (**b**) body surface of larvae of 2–3 mm, whose cuticle is still not divided in hexagonal tiles and the CaCO_3_ does not cover completely the cuticle but forms small aggregates scattered on the cuticle; (**c**) body surface of the larvae of 5 mm with a cuticle separated in hexagonal tiles. CaCO_3_ does not cover completely the cuticle but is localised at the centre of each tile forming three polygonal platelets in the centre; (**d**) prepupa body surface divided in hexagonal tiles fully covered by blocks of CaCO_3,_ which in their centre are crystallised in numerous juxtaposed polygonal platelets forming rosettes (arrows); (**e**) Puparium surface showing the same features of the prepupa; (**f**) prepupa body surface treated with HCl dissolving the blocks of CaCO_3_. Note the cuticle with its hexagonal tiles with a depression in their centre (arrow head).

**Figure 2:**
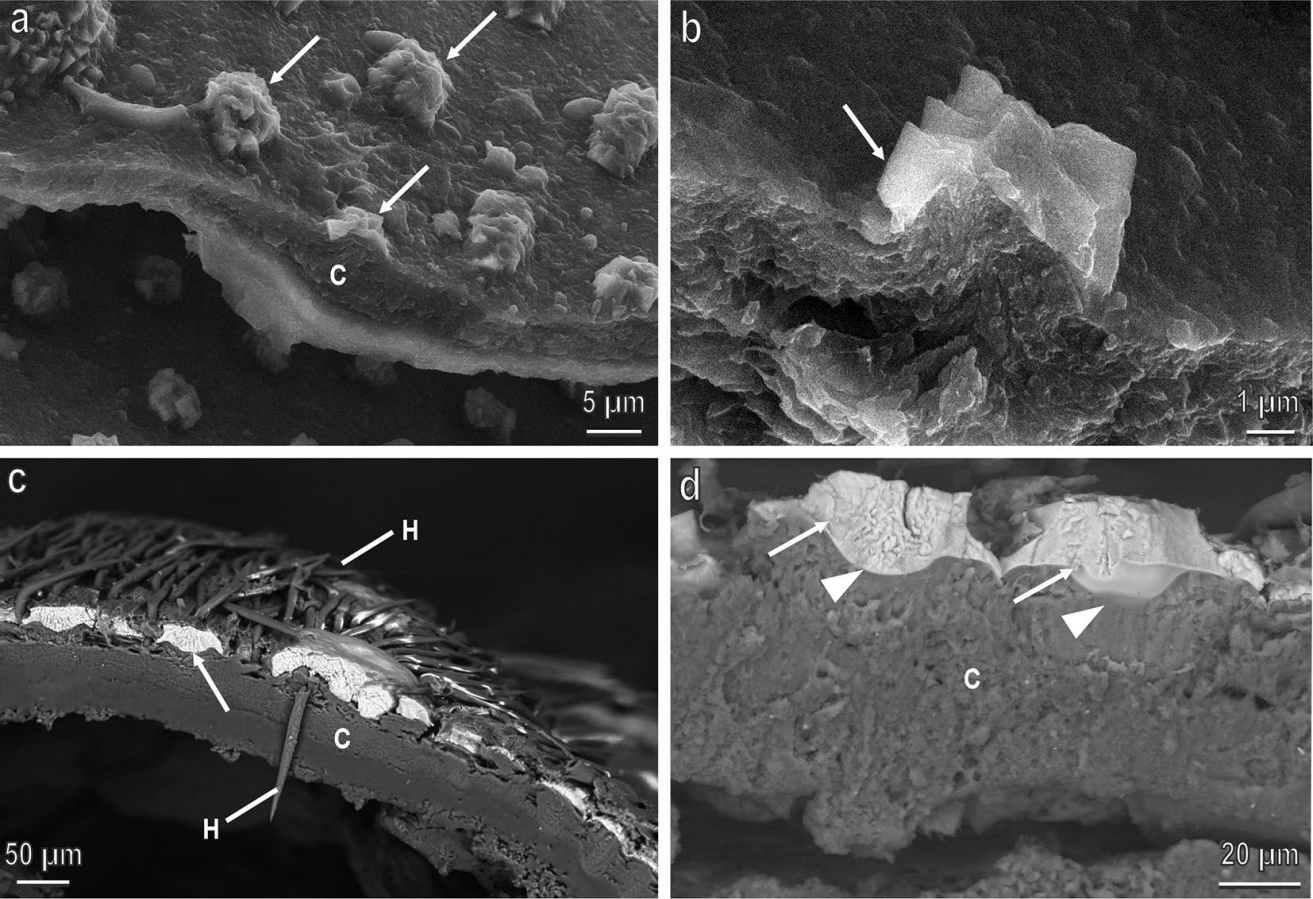
Cross sections of the cuticle of the larvae of 2–3 mm (**a**,**b**) and of the prepupa of *H. illucens* (**c**,**d**) at SEM (backscattered images) showing the CaCO_3_ blocks (arrows) in longitudinal section. In the young larvae the blocks are not developed and CaCO_3_ forms thin aggregates scattered on the cuticular surface while in the prepupa the CaCO_3_ blocks covers the hexagonal cuticular tile filling its central depression (arrow heads). A thick cuticle (C) underlies the CaCO_3_ blocks. H, hairs-like setae.

The cuticle of the larva of *H. illucens* in backscattered SEM images shows areas differing in intensity from the rest of the cuticle (Fig. 1b–e). Bright areas are already present in the young larval instars (Fig. 1b,c) but they reach maximum development in the prepupa instar (sixth instar) (Fig. 1d) where the body surface appears externally divided in hexagonal tiles fully covered by blocks which in their centre form numerous juxtaposed polygonal platelets forming rosettes. The cuticle of the prepupa is kept in the puparium which shows the same features (Fig. 1e). The treatment with HCl dissolves completely the blocks of the prepupa uncovering the cuticle with its hexagonal tiles and shows that each tile has a depression in its centre (Fig. 1f). The larvae of 2–3 mm have a cuticle which is still not divided in hexagonal tiles and the bright areas do not cover completely the cuticle but form small aggregates scattered on the cuticle (Fig. 1b). The larvae of 5 mm body length have a cuticle separated in hexagonal tiles and the bright areas do not cover completely the cuticle but are situated in the centre of each tile forming three polygonal platelets (Fig. 1c). Cross sections of the cuticle of the larvae of 2–3 mm body length (Fig. 2a,b) and of the prepupa (Fig. 2c,d) at SEM (backscattered images) show the blocks in longitudinal section; in both larval stages only the blocks are bright while all the rest of the cuticle underlying the blocks and the hairs emerging among the blocks are dark. In the young larvae, the blocks are not developed and the bright areas form thin aggregates about 2 µm thick and about 6 µm wide, scattered on the cuticular surface (Fig. 2b) while in the prepupa the blocks are about 20 µm thick and about 60 µm wide (Fig. 2d). Each block covers completely the hexagonal cuticular tile filling its central depression (Fig. 2d). A thick cuticle underlies the blocks (Fig. 2d).

**Figure 3:**
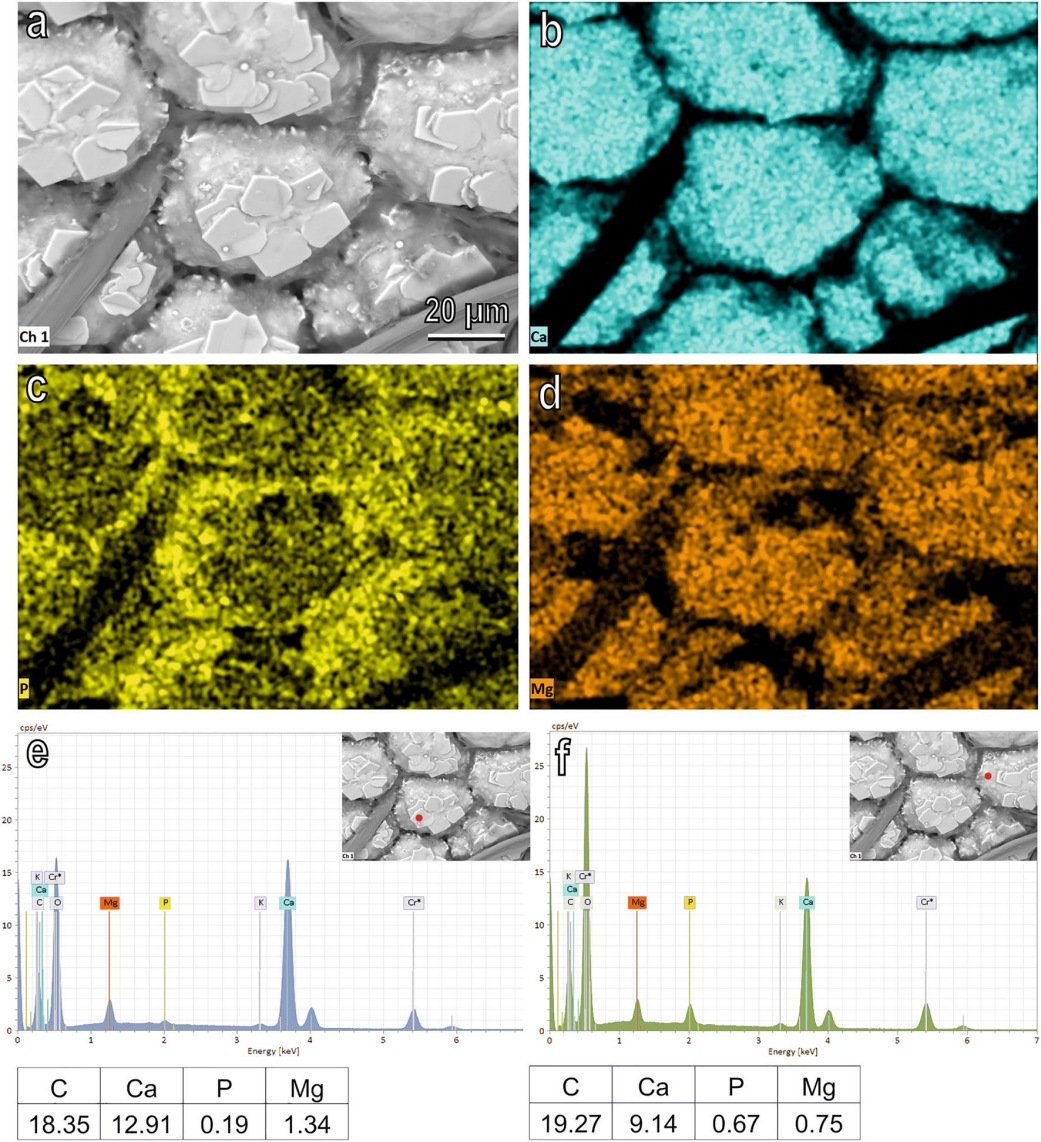
SEM image (**a**), spatial distribution of the most abundant elements represented by Ca (**b**), P (**c**) and Mg (**d**) and EDX analysis (**e**,**f**) on the body surface of the prepupa of *H. illucens*. Typical EDX spectrum (**e**,**f**) shows the presence of high amount of Ca (atomic percentage) both on the crystallised platelets forming rosettes on the blocks (**e**) and in the lateral portion of the blocks (**f**).

**Figure 4:**
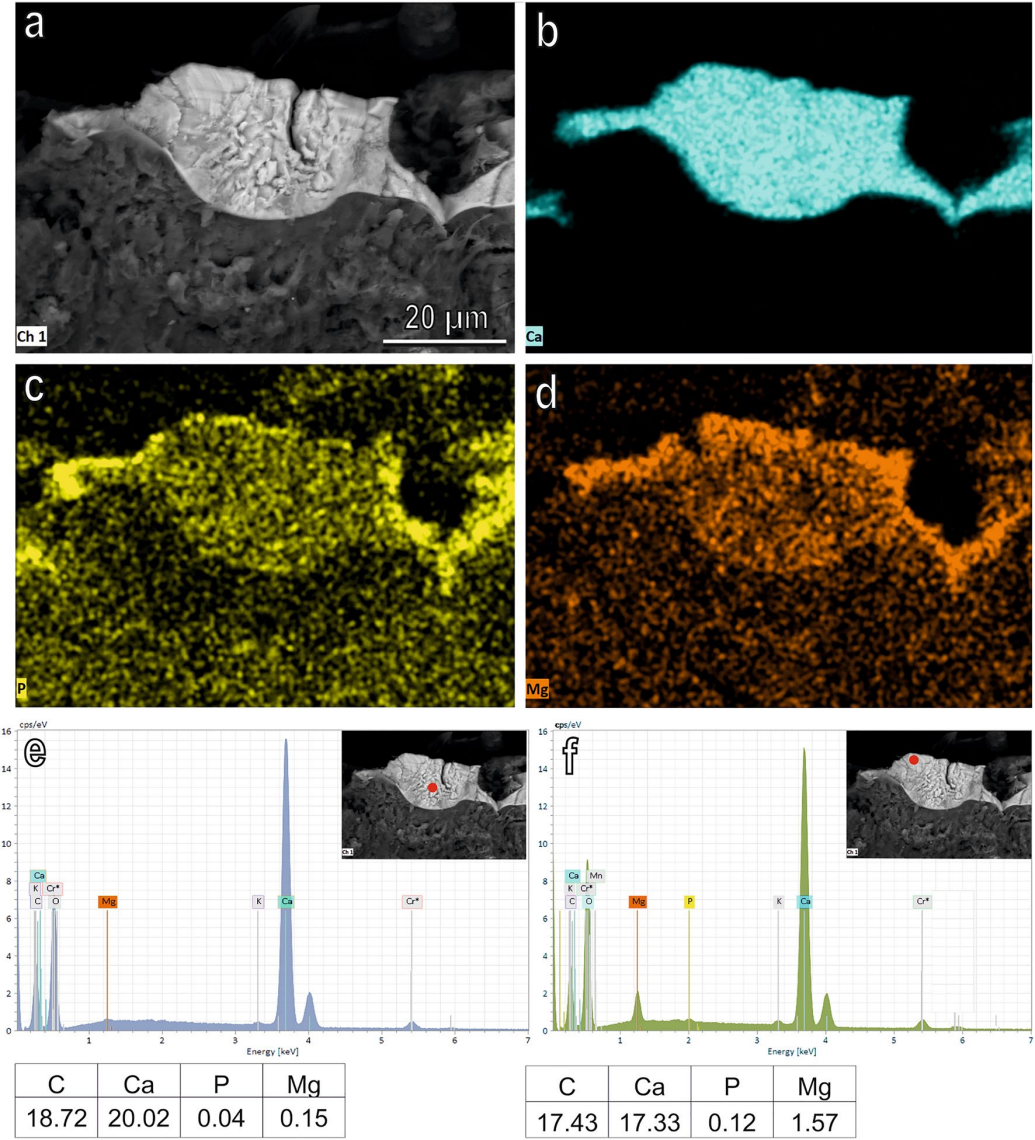
SEM image (**a**), spatial distribution of the most abundant elements represented by Ca (**b**), P (**c**) and Mg (**d**) and EDX analysis (**e**,**f**) on the prepupa blocks of *H. illucens* in longitudinal section. Typical EDX spectrum (**e**,**f**) shows that the amount of Ca (atomic percentage) is high compared with that of P and Mg both in the inner (**e**) and in the outer (**f**) portion of the block. A higher percentage of P and Mg is visible on the external portion of the block compared with its inner portion (**e**,**f**).

EDX microanalysis on the body surface of the prepupa (Fig. 3) and of the prepupa blocks in longitudinal section (Fig. 4) revealed that the most abundant elements (in addition to C, O and Cr used to metalize the specimens) are Ca, P and Mg. SEM images (Fig. 3a) and spatial distribution of Ca (Fig. 3b), P (Fig. 3c) and Mg (Fig. 3d) reveal that the amount of Ca (atomic percentage) is higher compared with that of P and Mg both on the crystallised platelets forming rosettes on the blocks (Fig. 3e) and in the lateral portion of the blocks (Fig. 3f). Mg seems more abundant than P in the crystallised platelets while in the lateral portion of the blocks P and Mg are present in equal proportions (Fig. 3e,f). SEM images (Fig. 4a) and spatial distribution of Ca (Fig. 4b), P (Fig. 4c) and Mg (Fig. 4d) on the prepupa blocks in longitudinal section reveal that the amount of Ca (atomic percentage) is more abundant compared with that of P and Mg both in the inner (Fig. 4e) and in the outer (Fig. 4f) portion of the block. A higher percentage of P and Mg is visible on the external portion of the block compared with its inner portion (Fig. 4e,f). The most abundant elements (in addition to C, O and Cr used to metalize the specimens) are Ca, P and Mg with Ca (atomic percentage) being more abundant compared with P and Mg also in the aggregates scattered on the body surface of the previous larval stages (Figs. SM1, SM2).

**Figure 5:**
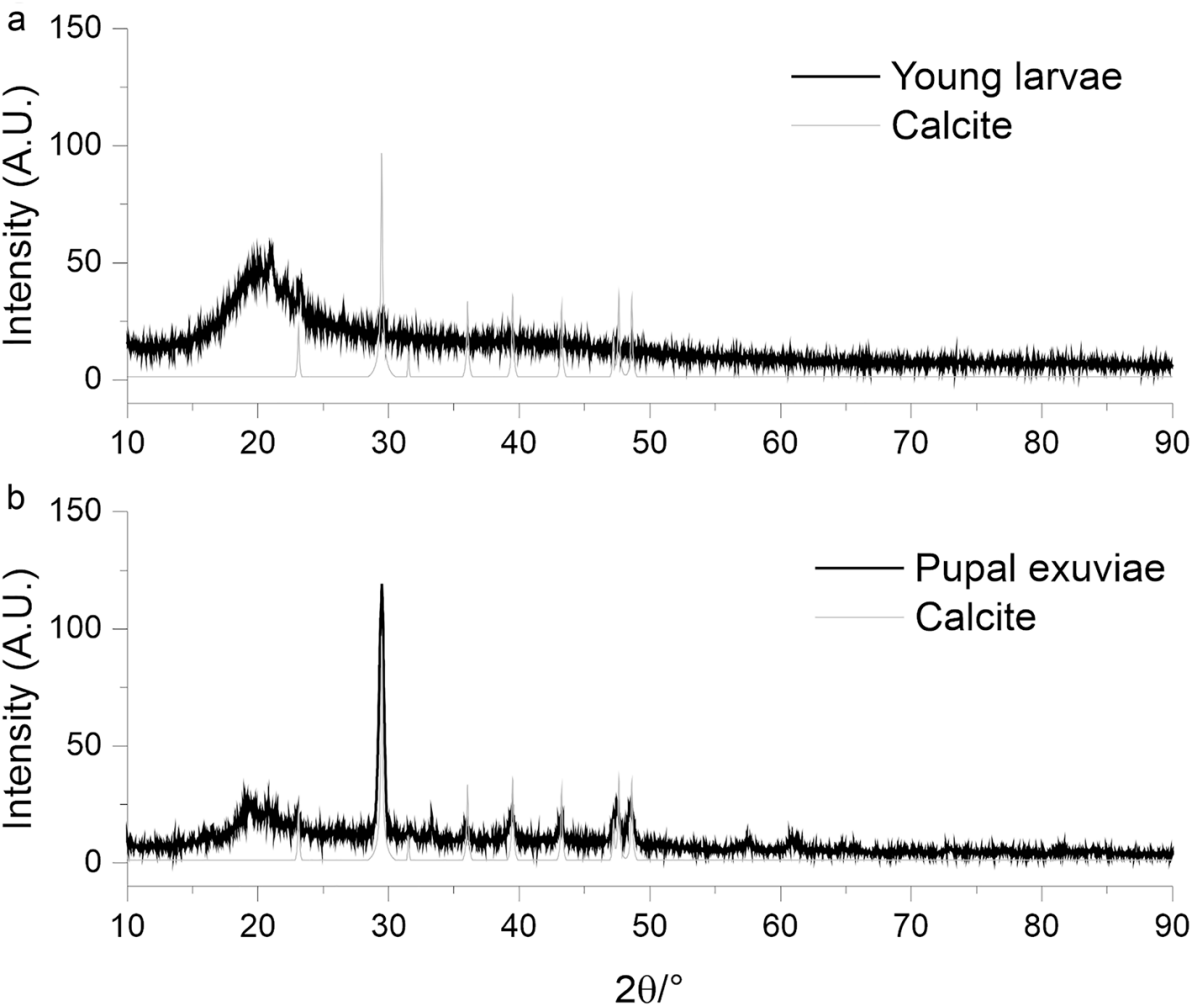
PXRD patterns collected on the young larvae (**a**), and the pupal exuviae (**b**) of BSF, the calculated one of calcite is reported in grey. In the young larvae the calcite typical reflections present a low intensity and broad peaks, due to the low crystallinity of the CaCO_3_ also observed in the SEM analysis. In the pupal exuviae of BSF sample, the PXRD pattern shows the presence of crystalline calcite.

In Fig. 5, the comparison between the observed PXRD patterns as well as the calculated calcite collected on the young larvae (Fig. 5a) and on the pupal exuviae of BSF (Fig. 5b) is reported. On the young larvae sample, an amorphous CaCO_3_ content can be associated with the broad reflection centred at 20°. In the pattern, the presence of 29.46° shows, as well, the presence of crystalline calcite in the young larvae. Furthermore, in the pupal exuviae of BSF sample, the crystalline calcite phase is evident, and the comparison between the calculated calcite pattern and the sample shows the overlapping between the reflections. A calcite crystal size of 31 nm was calculated using fundamental profiling. The increase in crystallinity in the two samples can be followed by observing the area of the main calcite reflection at 29.46°, with an increase of calcite content of about 76% in the pupal exuviae of BSF compared with larvae.

**Figure 6:**
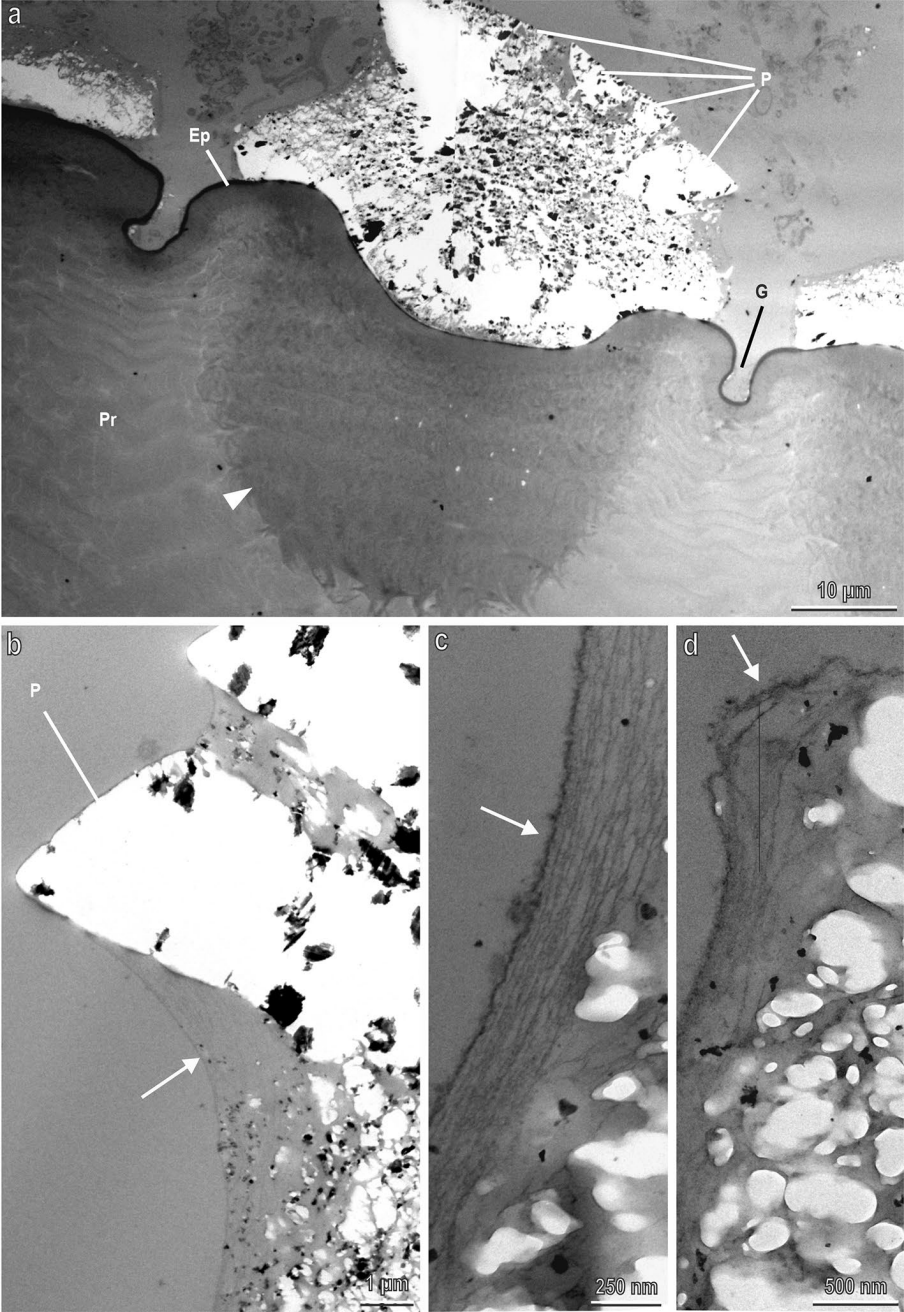
Sections of the cuticle of the prepupa of *H. illucens* in TEM. (**a**) Fine structure of a CaCO_3_ blocks with their crystallised platelets (P) and of the underlining cuticle. G, groove separating the tiles in the cuticle. Ep, epicuticle; Pr, procuticle. The procuticle is darker (melanised) in correspondence of the depression of each tile forming electron dense cones just under each CaCO_3_ block (arrow head). (**b**–**d**) Details of the blocks wrapped by a multi-layered membrane constituted of the most external epicuticular layers (arrow).

Sections of the cuticle of the prepupa observed in TEM reveal the fine structure of the blocks and that of the underlining cuticle (Fig. 6). Even if some part of the material in the blocks is not retained in the sections (empty white regions), probably lost during preparation or during sectioning, the shape of the blocks with the juxtaposed polygonal platelets is clearly visible (Fig. 6a). The groove, which separates each tile in the cuticle, is only few microns deep (Fig. 6a). The cuticle of the prepupa is constituted of an electron-dense epicuticle (thick about 2.5 µm), which is particularly evident in correspondence of the grooves among the blocks (Fig. 6a). Under the epicuticle the procuticle is visible (Fig. 6a). The procuticle is electron-dense in correspondence of the depression of each tile forming electron dense cones just under each block (Fig. 6a). The blocks and their apical platelets forming rosette are wrapped by a multi-layered membrane probably constituted of the most external epicuticular layers with a loose texture (Fig. 6b–d).

**Figure 7:**
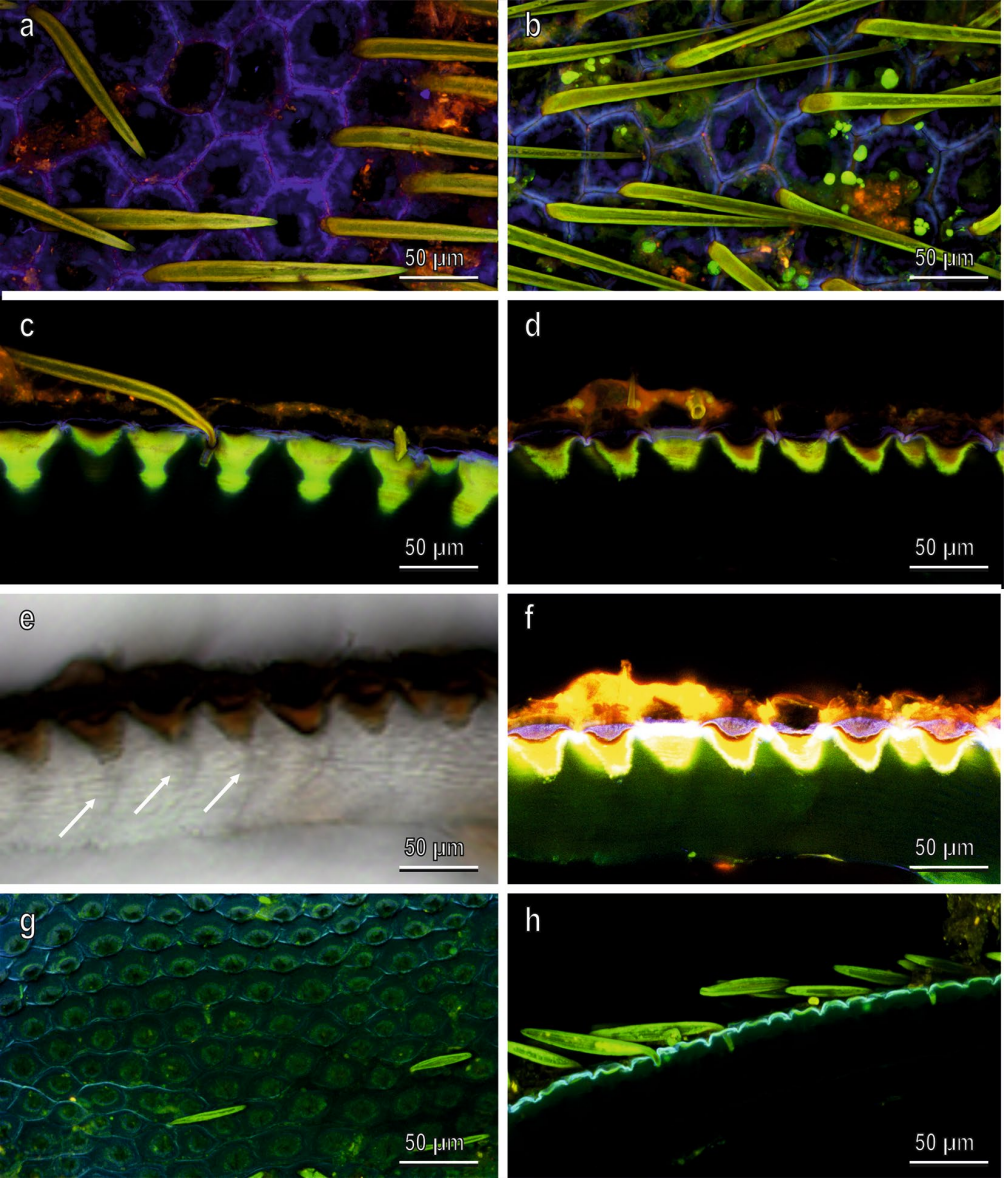
Confocal laser scanning micrographs of *H. illucens* cuticle. Prepupa cuticle, visualized from dorsal surface (**a**), ventral surface (**b**), section of the dorsal side (**c**) and section of the ventral side (**d**). The section of the dorsal side (**d**) was visualized by light microscopy (**e**), and increased in signal strength (**f**). Dorsal surface (**g**) and section of the dorsal side (**h**) of larva cuticle. Arrows point out the wide canals in the multilayered procuticle allowing the passage of CaCO_3_ granules (see Fig. 8).

Our observations of the prepupa body surface (dorsal and ventral side) in CLSM (Fig. 7) revealed that the outermost cuticle forming tiles hosting the blocks shows high intensities of the blue-coloured signal owing to the high amount of resilin, while hair cuticle is more sclerotized and appears green (Fig. 7a–d). The cuticular cones just under each block (which appear electron dense under TEM) show intense green colouration owing to strong autofluorescence, while the area surrounding the blocks is orange (Fig. 7c,d). The procuticle (which appears not pigmented in light microscopy, differently from the cones which appear brown, Fig. 7e) is dark in CLSM (Fig. 7c,d), but when the brightness and contrast of the image is increased, the weak signal of the procuticle is visible and appears mainly blue and slightly green (Fig. 7f). In the larva (clear larvae with a size of 10–15 mm, supposed to belong to the fifth instar), the cuticle surrounding the scale shows strong autofluorescence in blue (Fig. 7g,h) indicating that it may contain a large proportion of resilin, while the hairs are green as observed in the pupa.

**Figure 8:**
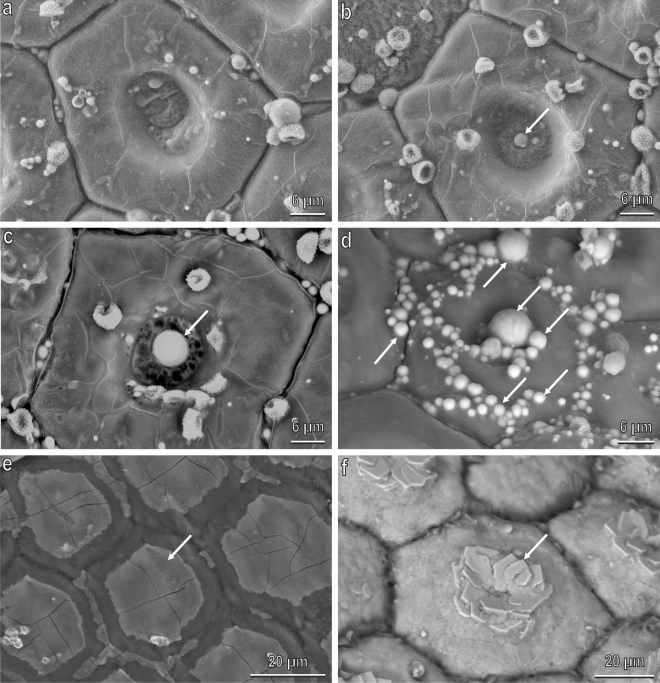
*H. illucens* prepupa after the moult in SEM (backscattered images). (**a**) just moulted larval body surface lacking the blocks of CaCO_3_; (**b**) In some hours after the moults, in correspondence of the central depression, ACC spherical aggregates appear (arrow); (**c**,**d**) the ACC spherical aggregates (arrows) increase gradually their size and number; (**e**) Encrustations of CaCO_3_ (arrow) covering the tiles; (**f**) Crystallised CaCO_3_ polygonal platelets (arrow) forming rosettes appear after about 48 h from the moult.

In the prepupa, the blocks fully developed on the larval body surface are visible only 48 h after the moult. In the just moulted prepupa (the sixth instar larva) (Fig. 8a), the larval body surface is similar to that described after treatment with HCl, lacking the blocks and showing its hexagonal tiles with the depression in their centre. In correspondence of the central depression, gradually after the moult, spherical aggregates appear (Fig. 8b), increasing gradually their size (Fig. 8c) and number (Fig. 8d). SEM images (Fig. SM3d) and spatial distribution of Ca, P and Mg (Fig. SM3e,f) of the spherical aggregates reveal a very high amount of Ca (atomic percentage). Gradually bright encrustations appear on the tiles (Fig. 8e) and finally, the polygonal platelets forming rosettes appear (Fig. 8f).

**Figure 9:**
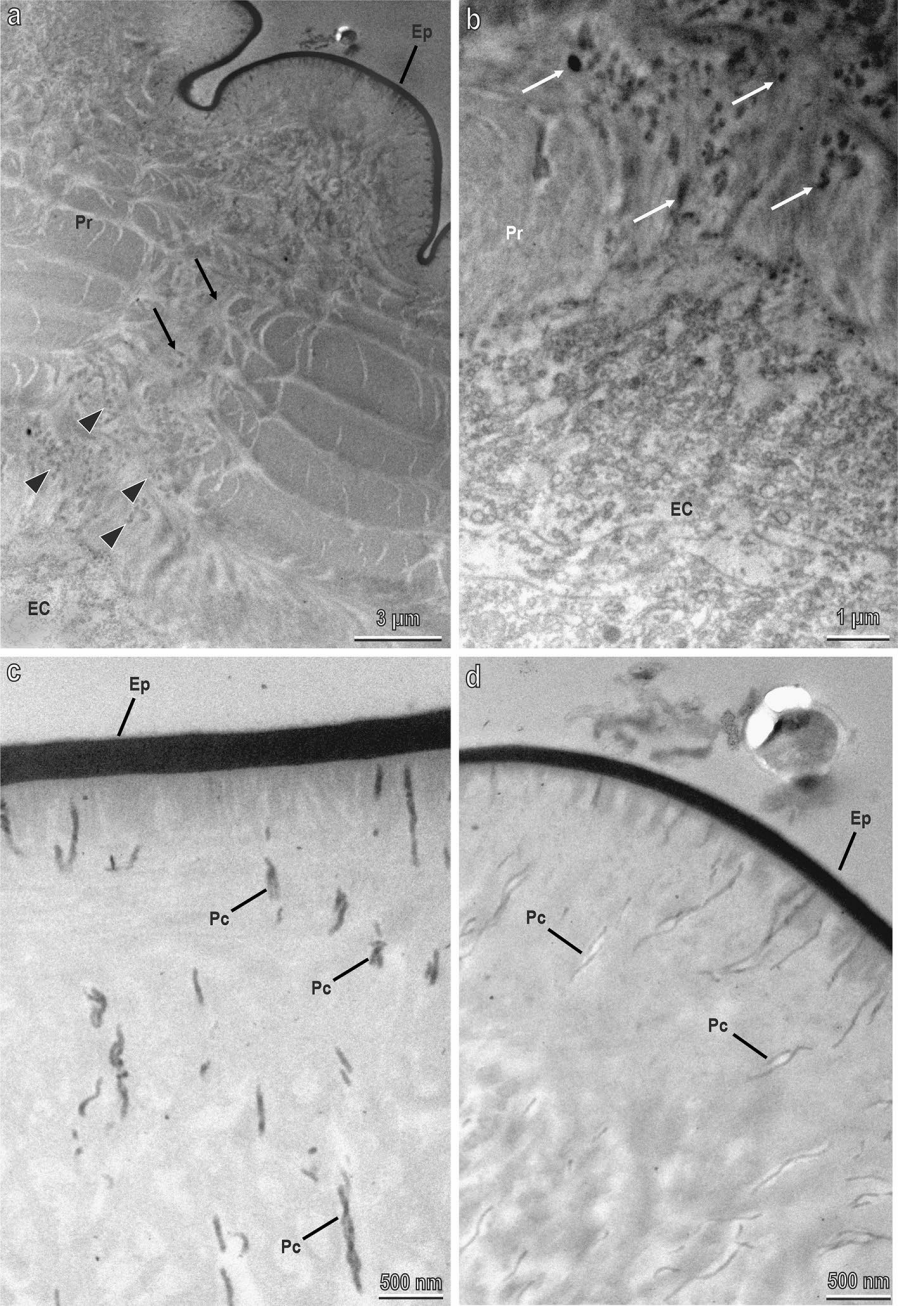
Sections of the just secreted cuticle of the fifth larval instar of *H. illucens* about to moult to the prepupa stage (the old cuticle has been removed) in TEM. (**a**) New multi-layered procuticle (Pr) under the central portion of each tile interrupted by wide canals (arrows) allowing the passage of dark granules (arrow head) from the epithelial cells (EC) to the epicuticular surface (E); (**b**) Detail of a); (**c**,**d**) Pore canals with dark secretions visible just under the epicuticular layer.

Sections of the just secreted new cuticle of the fifth larval instar (about to moult to the prepupa stage) observed at TEM (Fig. 9) reveal that under the central portion of each tile the new multi-layered procuticle is interrupted by wide canals (Fig. 9a), visible also in light microscopy (Fig. 7e), allowing the passage of dark granules from the epithelial cells to the epicuticular surface (Fig. 9b). Pore canals with dark secretions are visible just under the epicuticular layer (Fig. 9c,d).

**Figure 10:**
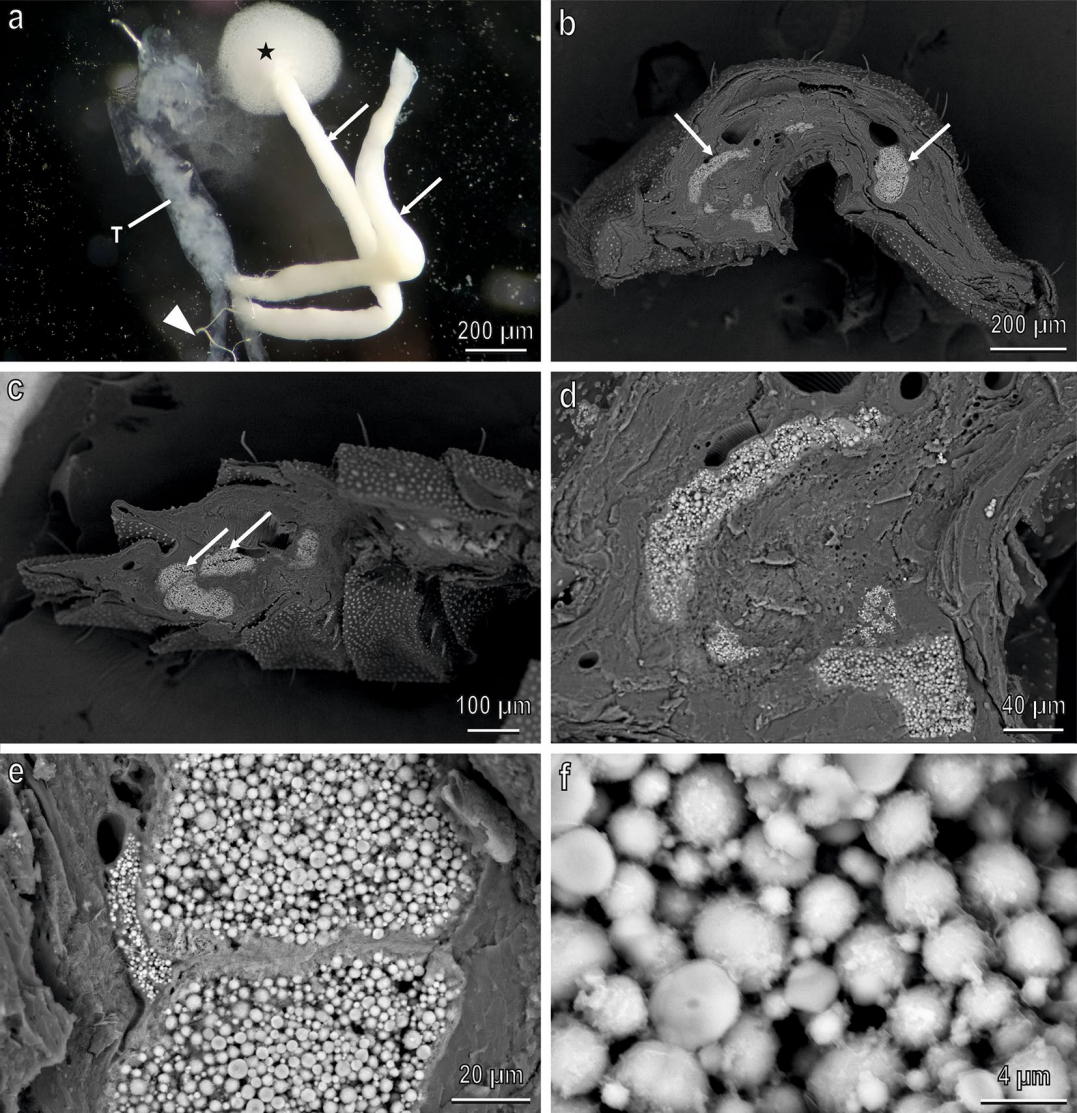
*H. illucens* (second instar larva) Malpighian tubules in light microscopy (**a**) and in SEM ((**b**–**e**), back scattered electrons. (**a**) Four Malpighian tubules emerging from a common trunk (T), two of them represent the real Malpighian tubules (arrow head) while the other two (white in colour) represent two storage organs of CaCO_3_ (arrows). Asterisk points their content made of lime; (**b**,**d**) Cross (**b**) and longitudinal (**c**) sections of dry larvae showing lime glands brighter compared with the surrounding structures, owing to presence of CaCO_3_ granules; (**e**,**f**) Details of (**b**) showing the CaCO_3_ spherical microgranules.

Observations using light microscopy of larvae belonging to different instars showed that *H. illucens* MTs emerge from a common trunk located at the transition between midgut and hindgut. From this trunk four long tubules emerge (Fig. 10a), two of them have the same morphology and colour of the common trunk and constitute the real MTs while the other two are bright white and, when broken, reveal their content made of white granules (Fig. 10a). Observations of cross (Fig. 10b,d) and longitudinal (Fig. 10c) sections of dry larvae in BSE SEM demonstrated compositional differences through contrast, showing MTs content brighter compared with the surrounding structures. The content of the two white MT is constituted of spherical microgranules with a diameter ranging from 1 to 5 µm (Fig. 10e,f). EDX microanalysis on these microgranules (Fig. SM4) revealed that the most abundant elements (in addition to C, O and Cr used to metalize the specimens) are Ca, P, Mg and K. Spatial distribution of Ca, P and Mg (Fig. SM4) reveals that the amount of calcium (atomic percentage) is high compared with that of P and Mg. The amount of P is relatively higher than that of Mg.

**Figure 11:**
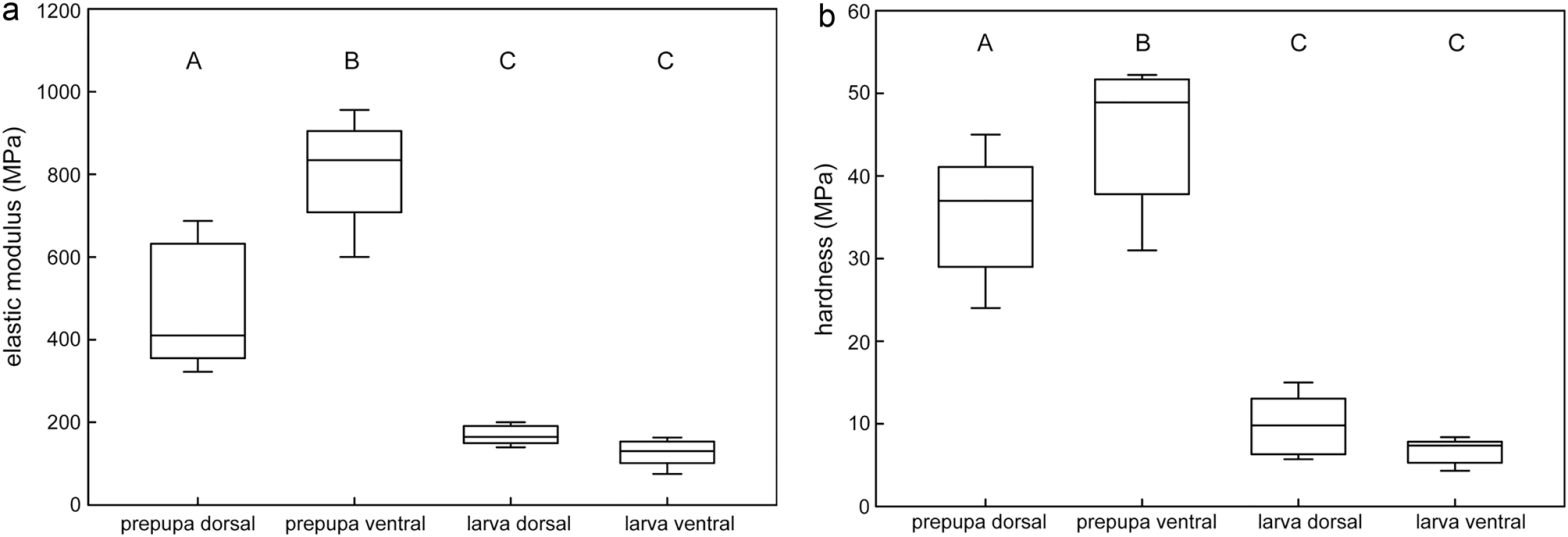
Mechanical properties of the cuticle of *H. illucens*. (**a**) Elastic modulus and (**b**) Hardness measured from the dorsal side of prepupa (PD, n = 5), the ventral side of prepupa (PV, n = 5), the dorsal side of larva (LD, n = 5) and the ventral side of the larva (LV, n = 5). Capital letters indicate significant differences (P < 0.05, Holm-Sidak one-way ANOVA).

We have measured the hardness and elastic moduli of the cuticle of larvae (clear larvae with a size of 10–15 mm, supposed to belong to the fifth instar) and of prepupa from both dorsal and ventral sides (Fig. 11). The hardness of the dorsal, ventral sides of prepupa and larva were equal to 35 ± 8 MPa, 46 ± 9 MPa, 10 ± 4 MPa and 7 ± 2 MPa (mean ± s.d), respectively, while the elastic moduli of the dorsal, ventral sides of prepupa and larva were 477 ± 151 MPa, 812 ± 130 MPa, 169 ± 24 MPa and 128 ± 32 MPa (mean ± s.d), respectively. Both hardness and elastic modulus are significantly higher in ventral side of the prepupa and lower in ventral and dorsal sides of the larva. Dorsal surface of the prepupa shows an intermediate value. For both hardness and elastic modulus, significant differences were found between every two groups except between dorsal and ventral side of larva (P < 0.05, Holm-Sidak one-way ANOVA).

## Discussion

### Calcite in the cuticle of *H*. *illucens*

In backscattered SEM images, in spite grey level could be influenced by topography of the sample^27^ (with brighter grey level than expected in more elevated portions of the samples), areas differing in intensity are generally related to different chemical composition with brighter areas composed of heavier elements. Indeed, in the present study, the EDX characterization of the pupal cuticle of the black soldier fly *H. illucens* indicates that the bright blocks with polygonal platelets forming rosette have a different chemical composition compared with the surrounding cuticle and contain a large amount of Ca and lower amounts of both Mg and P. The relative amount of different elements, observed in EDX analysis, is coherent with the presence of CaCO_3_, while there is no evidence of calcium phosphate, observed in the puparium of other Diptera^14,15^. Mg and P are present also in crustacean cuticle and could support ACC formation and stabilisation^5^. Mg seems more abundant than P in the crystallised platelets of BSF and this feature could be in agreement with the role of magnesium in the crystal growth of calcite^28^. Crystalline CaCO_3_ exists in three anhydrous forms represented by calcite, aragonite, and vaterite, with calcite representing the most stable polymorph of CaCO_3_ (even more stable than aragonite) under ambient conditions^29^. X-ray diffraction analysis on the pupal exuviae of the black soldier fly highlighted the presence of calcite, while the presence of vaterite or aragonite can be excluded. Moreover, a small amount of ACC is present. The presence of calcite is coherent with the shape of the polygonal platelets forming rosette on the surface of the blocks covering the cuticular hexagonal tiles of the pupa since calcite crystallizes in the hexagonal/rhombohedral space group^30^. Calcite is present in crustacean cuticle together with ACC^1,5^, but in these arthropods, we can observe mineralised exo- and endocuticle, because the α-chitin-protein organic fibres are associated with crystallites of both calcite and ACC, while in *H. illucens* pupa, calcite crystals are located in the epicuticle, wrapped by a multi-layered membrane constituted of the most external epicuticular layers, as appearing in our TEM observations. The involvement of epicuticular proteins in catalysing biomineralization and calcite crystallisation can be hypothesised in *H. illucens*, as recently demonstrated in the leaf-cutter ant *Acromyrmex echinatior* Forel (Hymenoptera: Formicidae), where a layer of magnesium-rich calcite overlaying the exoskeletons of workers is present^31^.

As it is clearly visible in the EDX analysis and in the x-ray diffraction analysis of young BSF larvae and the pupal exuvia, CaCO_3_ is already presented in the cuticle of young larval instars, but it is present mainly in the form of ACC, while the amount of calcite increases during larval development until the pupal stage. Moreover, the just-moulted larva totally lacks CaCO_3_, but gradually ACC spherical aggregates appear on the cuticular tiles increasing their size and number. Crystallization via amorphous precursor phases represents a general strategy in biomineralization and has been observed in different animal phyla, such as echinoderms (e.g. Ref.^32^), molluscs^33^, crustaceans^34^, annelids^35^ and chordates^36^. Mineral precursors of ACC can be conveniently stored during moult, re-dissolved, quickly manipulated into diverse shapes and being available after ecdysis. Amorphous particles aggregate, fill space, and crystallize by a mechanism of secondary nucleation^37^. In the fifth larval instar of *H. illucens* secreting new cuticle, dark granules are visible in TEM within the epithelial cells and inside the procuticle. The elemental composition of this material was not analysed in this study but, since in crustaceans pore canals play an important role in the transport of calcium^38^, we suppose that these granules could be ACC that move across the procuticle towards the epicuticular layer by means of pore canals.

### Storage organs

The storage organs of CaCO_3_ in *H. illucens* are represented by a pair of specialised MT which can be defined as “lime gland” according to their ability of CaCO_3_ accumulation. Their white appearance is related to their content represented by CaCO_3_ microgranules additionally containing P, Mg and K. We used the term “lime gland” for the specialised MT of *H. illucens* for their apparent similarity with those observed in the alkali fly *Ephydra hians* Say (Diptera: Ephydridae) inhabiting alkaline salt lakes containing extremely high concentrations of dissolved carbonate and bicarbonate^39^. In this case, the fly can regulate the high concentrations of environmental carbonate and bicarbonate through precipitation in the lime glands. In *H. illucens* larvae, the lime glands are presumably used as calcium storage deposits exactly as it happens in crustaceans (see reviews in Ref.^1,40^), where precipitates for temporary storage, such as gastroliths, and deposits in the ceca are typically composed of ACC (see review in Ref.^41^). ACC can be more easily dissolved than crystalline forms of calcium^42^ and in such form, calcium can be deposited relatively easily during ecdysis and newly dissolved after the moult to build up the new cuticle. We hypothesize a similar physiological adaptation in *H. illucens,* where EDX spectra show the presence of a relatively low amount of Ca on a just shed exuvia (Figs. SM3a–c) and a higher amount on the body surface of the larva few hours after the moult (Figs. SM3d–f), but further investigations are necessary to clarify this aspect.

Among the different functions of insect MT (review in Ref.^43^), their involvement as storage organs of inorganic salts (although not so developed as in *H. illucens*) has been highlighted in other Diptera species, such as *M. autumnalis*, where spherical granules of minerals with a diameter of 0.2 to 10.0 μm are formed and stored in the distal region of the anterior pair of MT^44–46^ to be used to harden the pupal cuticle. A different pH in the different regions of the MT has been suggested to be involved in the dissolution and release in the haemolymph or accumulation of these granules^47,48^. A relatively high P/Ca ratio of about 0.14 (Fig. SM4) could suggest storage in MT of a small amount of calcium phosphate in addition to calcium carbonate but the EDX microanalysis of the blocks is not coherent with the presence of this mineral in the cuticle of *H. illucens*. More probably, P ions could participate in the stabilization of the amorphous phase of calcium carbonate, as suggested for different organisms (Kababy et al.^49^).

While marine crustaceans replace calcium across their gills from the seawater which contains a high amount of this element, terrestrial crustaceans, like isopods, depend on calcium obtained with their diet and, in this regard, they developed the largest calcium reservoirs observed in Crustacea, represented by the sternal plate^4^. Storage of ACC and its resorption are highly regulated during moulting in *Porcellio scaber* Latreille (Isopoda: Porcellionidae)^3^. Isopod sternal plates and the white lime glands of *H. illucens* could represent analogous storage structures which can be regarded as an adaptation to calcium deficient environments^2^, revealing convergent evolution between crustaceans and insects.

### Mechanical properties of the larval cuticle of *H*. *illucens*

CLSM observations on the cuticle of larvae and pupae of the BSF revealed that the cuticle hosting the blocks shows a high amount of the elastic protein resilin at both stages. Resilin is a mechanically highly deformable protein showing almost perfect elastic recovery^50^. It is widespread in arthropod cuticle (see review in Ref.^51^) and is important for the flexibility of arthrodial membranes and joint systems, storage of elastic energy, adaptability to uneven surfaces in attachment, etc. Its presence in the young stages of the BSF guarantees cuticle flexibility necessary for larval locomotion on the substrate, such as rotten material and for prepupal locomotion, when the larva stops feeding and moves to seek shelter to pupate^52^. On the other hand, the presence of CaCO_3_ blocks reduces elasticity but hardens the cuticle as clearly demonstrated in our indentation measurements. Young’s modulus of the resilin is about 1 MPa and the mean value for insect larval cuticle is about 10 MPa^11^, while *H. illucens* Young’s modulus reaches 169 MPa on dorsal side of the larva and in the prepupa, where the CaCO_3_ blocks are fully developed, it reaches 812 MPa on the ventral side. In parallel, cuticle hardness increases ranging from 10 MPa in the larva to 46 MPa in the prepupa. These data demonstrate the importance of calcite blocks to harden the cuticle in the BSF even more efficiently to what has been observed in the leaf-cutter ant *A. echinatior*^31^, where the layer of magnesium rich calcite overlaying the exoskeletons allows obtaining a composite structure with a two-fold increase in hardness compared with simple cuticle. At the moment we do not know the reasons of the differences in elasticity and hardness between the ventral and dorsal side of the pupal cuticle and this aspect needs further investigation.

### Function of the armoured cuticle of *H*. *illucens*

The presence of cuticle armoured with calcite is unique to the Stratiomyomorpha and, therefore, supports the monophyly of the infraorder^16,17^. In consideration that some subfamilies of Stratiomyidae are terrestrial and live in leaf litters or in the soil with decaying matter represented by animal or vegetal debris, they can be subjected to considerable abrasion and they need a thick and robust cuticle where calcite blocks and resilin-enriched cuticle can guarantee hardness, durability and flexibility. As above reported, hemispherical protrusions of amorphous calcium phosphate have been described on the epicuticle of other soil-dwelling dipteran larvae, such as the fly *E. angustifrons*^15^. The presence of aragonite in the cuticle of the beetle *Eudicella gralli* (Buquet) (Coleoptera: Scarabaeidae)^53^ is in agreement with the function of the dorsal protrusion of this scarab as a ‘shovel-like’ device, which females use to dig through the wood. On the other hand, the calcite biomineral layer of BSF can be used also as protective armour by immobile BSF pupae which can be subjected to predation. Indeed, in the leaf-cutter ant *A. echinatior*^31^, the layer of magnesium-rich calcite overlaying the exoskeletons allows higher survival for workers when fighting with other ants. A further function of calcite armour could be related to pathogen protection, demonstrated as well in the calcite armoured ant *A. echinatior*, when exposed to an entomopathogenic fungus^31^. Habitats of BSF larvae are characterized by an abundance of microorganisms and potential pathogens and for this reason, they can produce antibacterial peptides^54^. The calcite-enriched cuticle can represent a further defence against bacteria, fungi, and viruses.

## Conclusions

In relation to their great diversification, insects developed different cuticle ’configurations’ with various material and mechanical properties to adapt to different environments^55^. In spite of the widespread presence of calcium carbonate in the crustacean cuticle, biomineralisation in insects, deriving from Crustacea^56^, has so far remained unknown^31^. In the present study, we investigated in detail the morphology and material properties of the cuticle of BSF larvae. In this regard, we believe that *H. illucens* could represent a model species, to study biomineralisation in this taxon and further physiological and morphological investigations could deepen our knowledge regarding the ability of these insects to accumulate and store CaCO_3_, to precipitate and crystallise it in the form of calcite and to use it to decorate and strengthen their cuticle. In spite of the great interest concerning this insect species in the circular economy, many basic aspects concerning *H. illucens* biology remain so far uninvestigated and increased knowledge regarding cuticle biomineralization in BSF larvae could also improve their use as mineral source in animal diet. Lastly, our study not only adds important information to unravel insect biomineralization but also can potentially provide ideas for the design of novel biomimetic and synthetic materials.

## Methods

### Insect rearing

Pupae of BSF bought from Azienda Agricola Demetra Srls (Palmi, Italia) were kept inside a wood and Plexiglas cage (50 × 50 × 50 mm) in a controlled condition chamber (14 h photoperiod, temperature of 28 ± 3 °C, and relative humidity of 60 ± 10%) until adult emergence. The emerged males and females were kept in the same cage for mating under a LED light (STASUN, 9000 lm, 100 W, 5000 Kelvin degrees) and were provided with water and crystallised sucrose. Ovipositing females were provided with plastic cylindrical pots (7 cm in diameter, 8 cm high) containing the Gainesville diet (50% wheat bran, 30% alfalfa meal, and 20% corn in weight) diluted in water. The lid of the pots was perforated and squares of cardboard were inserted in it, in order to provide oviposition sites for the females. The pots were checked every day and the cardboard with just laid eggs was removed and placed in other pots containing wet paper, in order to keep moisture for egg hatching. After three days, the emerged first instar larvae were moved with a small brush to other transparent plastic containers (16 × 14 × 5 cm^3^) with the lid replaced with a mesh, containing the Gainesville diet added with water (125 cc of water in 100 gr of diet) for larval development. The pupae were moved to the above reported wood and Plexiglas cage (50 × 50 × 50 mm) for adult emergence. Considering that BSF larval development takes place in 6 instars^57–59^ characterised by a very variable size in each instar^60^, we used for experiments (Fig. 1a) larvae with a size of 2–3 mm (supposed to belong to the second/third instar), larvae with a size of 5 mm (supposed to belong to the third/fourth instar), clear larvae with a size of 10–15 mm (supposed to belong to the fifth instar) and dark larvae with a size of 10–15 mm (belonging to the sixth instar, also called prepupa). These last larvae keep their cuticle as puparium and become immobile pupae at the end of the larval development.

Some specimens were kept isolated until the moult to observe their cuticle just after the moult.

### Light microscopy

To describe the MTs of BSF larvae, we performed observations under a stereomicroscope Leica MZ6 (Leica Microsystem GmbH, Wetzlar, Germany).

### Scanning electron microscopy (SEM)

Larvae and pupae were anaesthetized with carbon dioxide, frozen and dried in an oven at 40 °C for 48 h. Some pupae were treated with HCl 37% (Roth) for 1 min and then were washed with distilled water. Afterwards, samples (whole larvae and portions/sections of larvae and pupae and HCl treated pupae) were mounted on aluminum stubs using double-sided carbon tape and metalized with a thin layer of chromium (8 nm). Some larvae were cut (after being mounted on the stubs) in longitudinal and cross section using a razon blade to observe the cuticle in section and the internal content of the body (in particular, MTs). The morphology of the cuticle and of MTs was analyzed by field emission scanning electron microscopy FE SEM LEO 1525 (ZEISS) using backscattered electrons (BSE) at 15 kV and secondary electrons at 5 kV. Backscattered SEM images demonstrate compositional differences through contrast (brighter areas indicate the presence of higher atomic number composition material).

### Scanning electron microscopy with energy dispersive X-ray microanalysis (EDX)

Larvae and pupae were prepared as for SEM observations. The quantitative element composition in the cuticle and in the MTs of *H. illucens* was determined using a Field Emission Scanning Electron Microscope (FE-SEM) LEO 1525 ZIESS equipped with an energy-dispersive x-ray detector (EDX) (Bruker Quantax). Following parameters were applied: measurement time 5 min, accelerating voltage 15.00 kV, working distance 9 mm.

### Transmission electron microscopy (TEM)

The larvae and pupae were cut in small portions along the transversal plane and fixed for 3 h in 2.5% glutaraldehyde in sodium cacodylate buffer (Electron Microscopy Sciences, Hatfield, PA, USA) with a pH of 7.2, then were repeatedly rinsed in sodium cacodylate buffer and post-fixed for 1 h at 4 °C in 1% osmium tetroxide in sodium cacodylate buffer (Electron Microscopy Sciences). The samples were then repeatedly rinsed in the same buffer, dehydrated by using ascending ethanol concentrations and finally embedded in an Epon-Araldite resin mixture (Sigma-Aldrich). Afterwards, ultra-thin sections were cut using a Leica EM UC6 ultramicrotome (Leica Microsystem GmbH, Wetzlar, Germany), collected on Formvar (Sigma-Aldrich) coated copper grids, stained with uranyl acetate and lead citrate (Electron Microscopy Sciences) and examined with a Philips EM 208 TEM (Philips, Eindhoven, the Netherlands). Digital pictures were obtained using a high resolution digital camera MegaViewIII (SIS) connected to the TEM, with an analysis software (Olympus Soft Imaging, Germany).

### Confocal laser scanning microscopy (CLSM)

A CLSM-based method established by^61^ to analyse material compositions and their gradients in arthropod cuticle by visualizing autofluorescences was applied. We interpreted the final images and described the material properties of cuticle as followings: (1) red colored areas are likely well-sclerotized, (2) green to yellow colored areas are less sclerotized in comparison to red colored ones and are mechanically stable but relatively flexible due to the presence of less sclerotization, and (3) blue colored areas are rubber-like with a relatively high proportion of resilin-like proteins. This method was already applied to many arthropod structures^62–64^.

Larvae and prepupae of BSF were frozen in a conventional lab freezer (ca. − 20 °C) for 10 min. The pieces of fresh cuticle were cut from the larvae and prepupae of BSF by a scalpel. Subsequently, transverse sections with a thickness of ~ 300 μm were made using a sharp razor blade. The specimens were washed in 75% ethanol and then immersed in glycerine (≥ 99.5%, Carl Roth GmbH & Co. KG, Karlsruhe, Germany). After fixing the specimens in glycerine between a glass slide and a cover slip (Carl Roth GmbH & Co. KG, Karlsruhe, Germany) for ~ 2 h, we visualized them with the CLSM (Zeiss LSM 700, Carl Zeiss Microscopy, Jena, Germany). The CLSM was equipped with four lasers (laser lines: 405 nm, 488 nm, 555 nm, 639 nm) to excite the sample fluorescences subsequently. Four emission filters transmitting 420–480 nm, ≥ 490 nm, ≥ 560 nm and ≥ 640 nm were used to visualize different fluorescences of the cuticle components. We have visualized the dorsal and ventral cuticle from both larva and prepupa of BSF. In total, 8 transverse sections from 4 individuals were scanned.

### Powder X-ray diffraction (PXRD)

A sample of 20 pupal exuviae of BSF and a sample of younger larvae (supposed to belong to the third, fourth and fifth instars) were analysed. Whole pupae and larvae were frozen and dried for 24 h in the oven at 60 °C, were ground in an agate mortar, in order to obtain two homogeneous samples (one with pupae and one with larvae). Powder X-ray diffraction (PXRD) patterns were collected in reflection geometry in the 5–90° 2θ range, with a 100 s step^–1^ counting time and with a step size of 0.008°on a Bruker D8 Advance diffractometer (Bruker Corporation, Billerica, MA), D8 goniometer, (Bruker Corporation, Billerica, MA) equipped with an LYNXEYE XE-T detector (Bruker Corporation, Billerica, MA) by using the Cu Kα radiation. The long fine focus (LFF) ceramic tube operated at 40 kV and 40 mA. Crystal sizes, calculated with TOPAS 4 (Bruker software), were obtained with Rietveld refinement in the pattern range 25° 80° using the fundament profiling approach and refining the crystal size.

### Nanoindentation measurements

Pieces of ~ 3.0 × 3.0 × 0.1 mm^3^ size fresh cuticle were dissected from both dorsal and ventral sides of the larvae and prepupae of BSF using a razor blade. They were fixed on a specimen holder, within 2 min after the dissection, using super glue (5925 Ergo, Kisling AG, Wetzikon, Switzerland). To minimize the desiccation during measurements, we have then used the wet cotton, to surround the specimens, and parafilm (BEMIS Packaging Deutschland GmbH, Rheinbach, Germany), to additionally cover the wet cotton and specimens except for the measurement area^65^. The hairs on the measurement area were carefully removed with a fine tweezer. The specimens were indented using an SA2 Nanoindenter (MTS Nano Instruments, Oak Ridge, Tenn., USA) equipped with a Berkovich diamond tip. The elastic modulus (Young’s modulus, the ratio of the force per unit area applied to the object and the resulting deformation in the linear elastic region of the material) and hardness of the specimens were measured using continuous stiffness measurement (CSM) technique. Nanoindentations were performed on ten sites on each sample with the distance between adjacent indentation sites on the surface of each specimen ≥ 40 μm, to avoid interference between consecutive measurements. The maximum indentation depth was set as 2 μm, while the strain rate, harmonic displacement and harmonic frequency were set as 0.05 s^–1^, 1.0 nm and 75 Hz. Poisson’s ratio of specimens was assumed to be 0.3^66^. The thickness of the dorsal cuticle was 60 ~ 70 µm, while that of the ventral cuticle was 80 ~ 90 µm. The indentation depth was 2 µm. Therefore, in both cases, the ratio of indentation depth to cuticle thickness was < 10%. In total, 20 samples taken from 10 individuals were measured.

### Supplementary Information

Supplementary Figures.

## Data Availability

Data are available from the corresponding author on request.
