# Supplementary material for: The armoured cuticle of the black soldier fly Hermetia illucens
Source: Sci Rep. 2023 Dec 13;13:22101. doi: 10.1038/s41598-023-49549-5 (PMC10719276; doi:10.1038/s41598-023-49549-5)
Supplement: Supplementary file 1 — Supplementary Figures. [file 41598_2023_49549_MOESM1_ESM.pdf]

Supplementary figures

Calcite in the cuticle of the black soldier fly *Hermetia illucens*

Manuela Rebora [1], Gianandrea Salerno [2]*, Silvana Piersanti [1], Valerio Saitta [2], Diletta Morelli Venturi [1], Chuchu Li [3], Stanislav Gorb [3]*

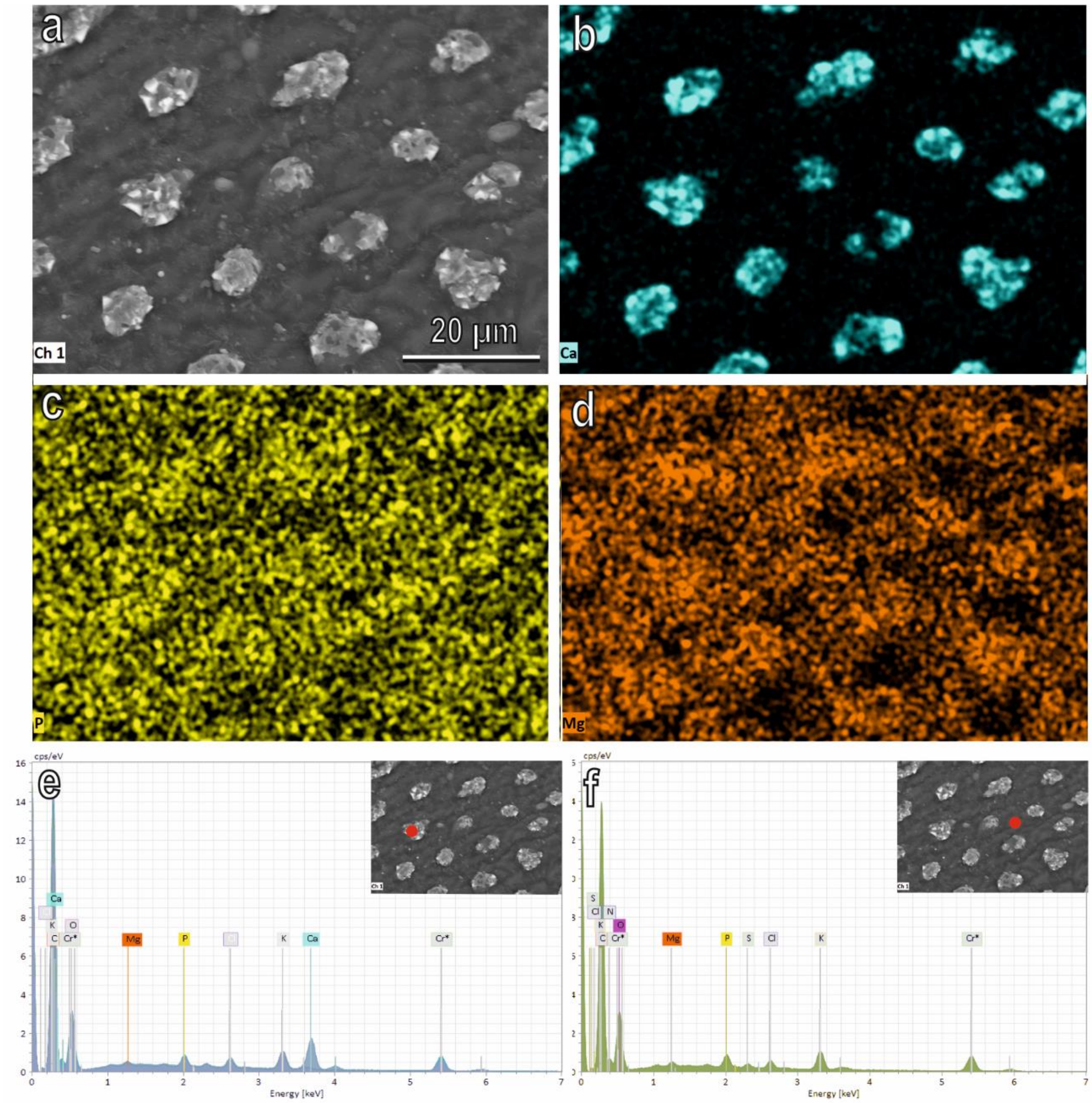

C	Ca	P	Mg
70.47	2.13	0.30	0.12

C	Ca	P	Mg
61.62	-	0.46	0.16

Fig. SM1. SEM image (a), spatial distribution of the most abundant elements represented by Ca (b), P(c) and Mg (d) and EDX analysis (e,f) on the body surface of the young larvae of 2-3 mm (supposed to belong to the second/third instar) of *H. illucens*. Typical EDX spectrum on the granules (e) and on the cuticular surface (f) shows the presence of a low amount of Ca (atomic percentage) concentrated in the granules and traces of P and Mg visible in the aggregates and also on the cuticular surface.

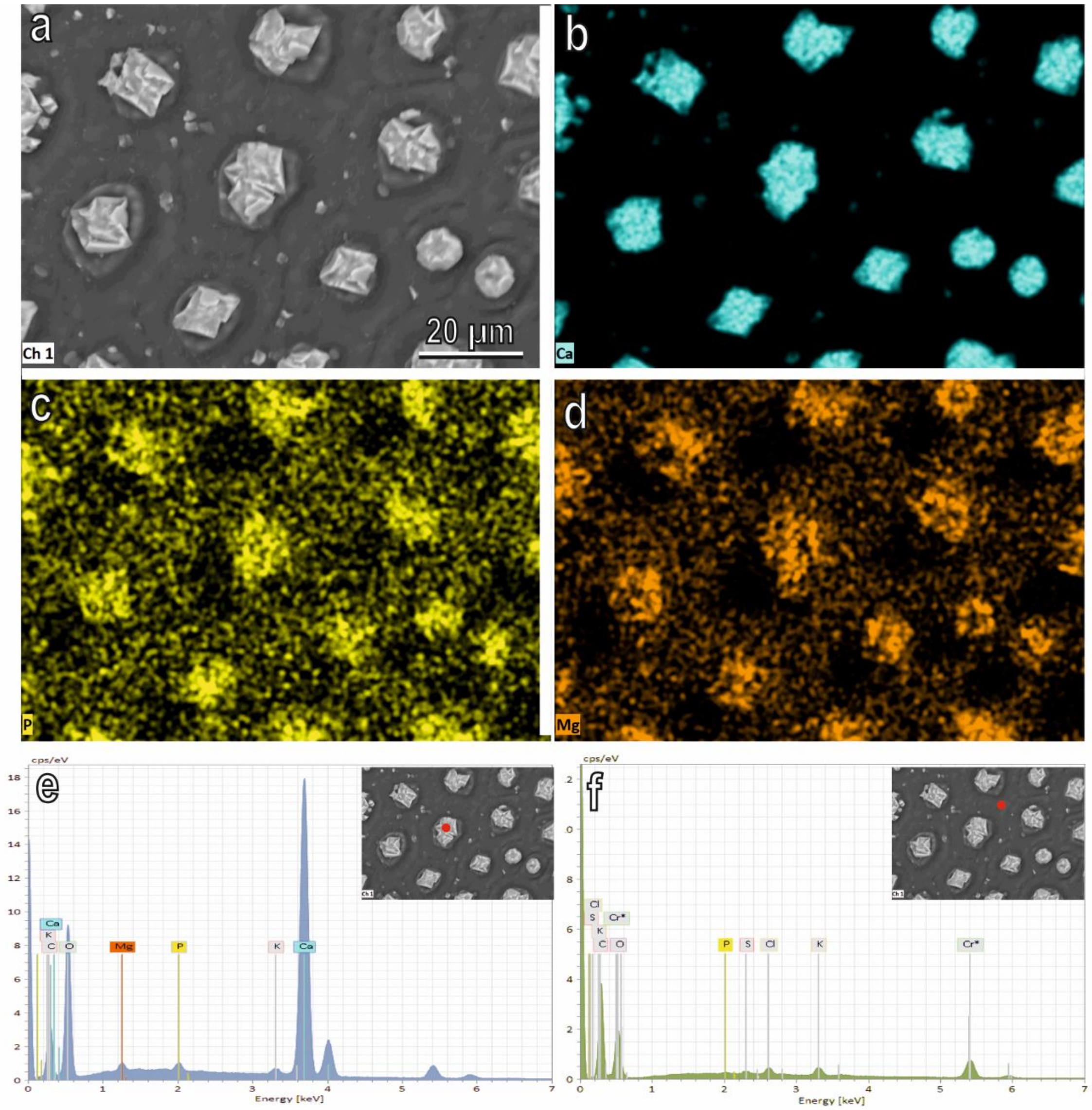

C	Ca	P	Mg
17.17	19.09	0.31	0.45

C	Ca	P	Mg
59.21	-	0.15	0.16

Fig. SM2. SEM image (a), spatial distribution of the most abundant elements represented by Ca (b), P(c) and Mg (d) and EDX analysis (e,f) on the body surface of the larvae of 5 mm (supposed to belong to the third/fourth instar) of *H. illucens*. $CaCO_3$ aggregates do not cover completely the cuticle but are localised at the centre of each tile forming three polygonal platelets. Typical EDX spectrum **(e,f)** shows the presence of high amount of Ca and traces of P and Mg (atomic percentage) in correspondence of the platelets. On the cuticular surface Ca is absent and a lower amount of P and Mg in comparison with the $CaCO_3$ aggregates is present.

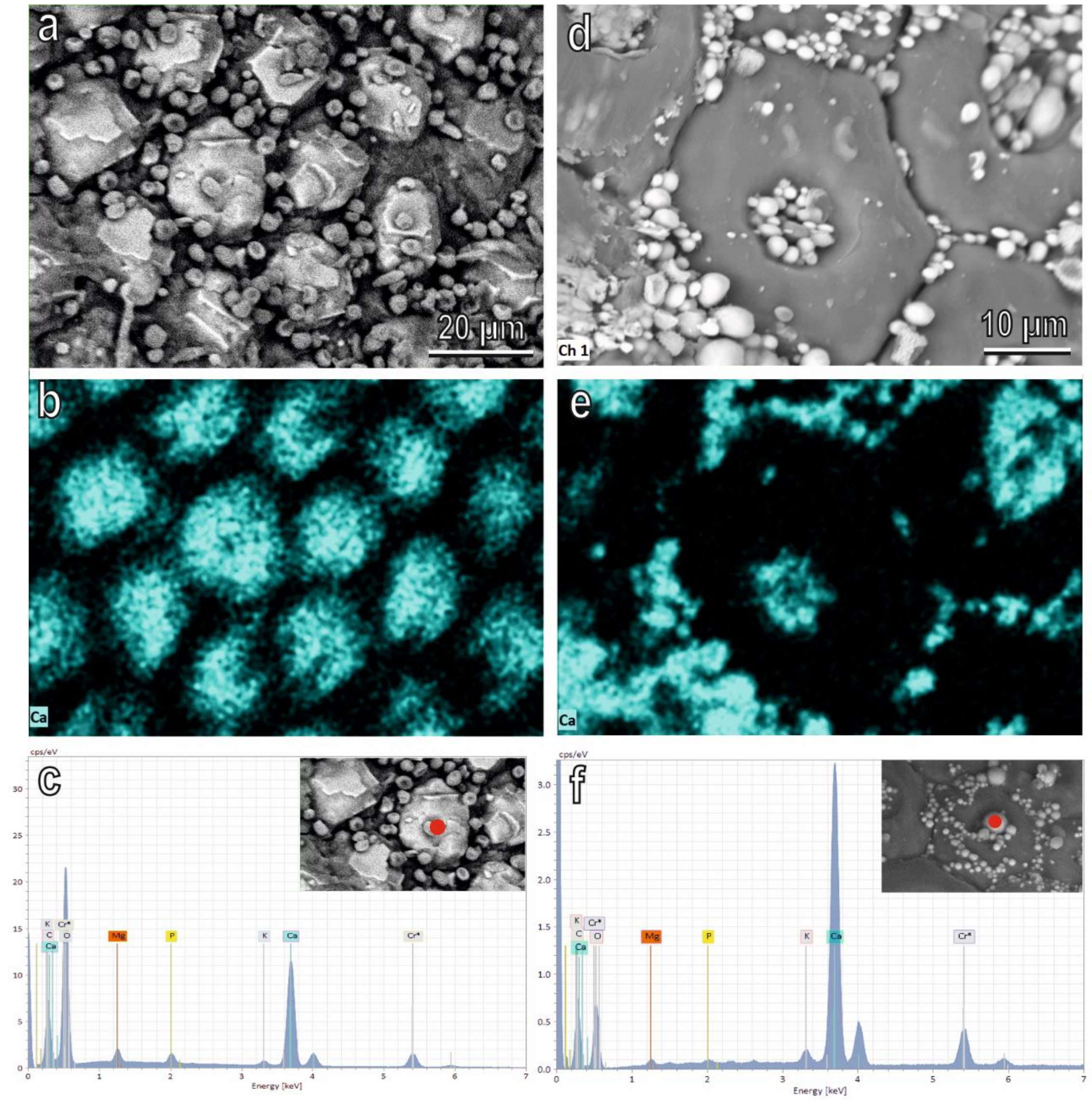

C	Ca	P	Mg
21.21	8.70	0.43	0.53

C	Ca	P	Mg
25.34	25.48	0.26	0.84

Fig. SM3. SEM image (a), Ca spatial distribution (b) and EDX analysis (c) of the exuvia of a larva of *H. illucens* moulting from the fifth to the sixth instar and SEM image (d), Ca spatial distribution (e) and EDX analysis (f) on the body surface of the same larva few hours after the moult. Typical EDX spectrum shows the presence of a certain amount of Ca (atomic percentage) in correspondence of the platelets of the exuvia **(c)** and of a very high amount of calcium in correspondence of the aggregates of amorphous calcium carbonate accumulating on the cuticular surface after the moult **(f).**

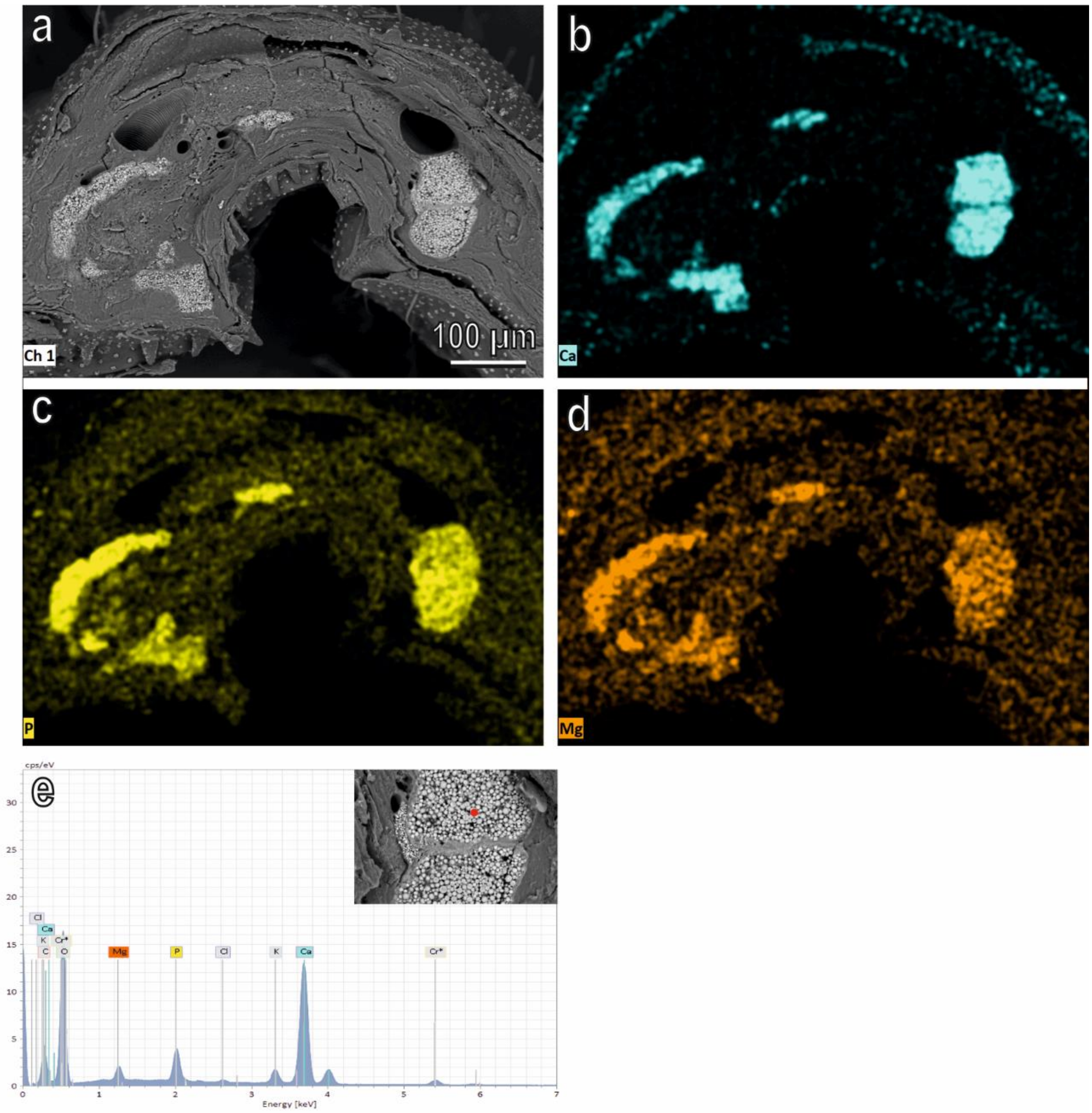

C	Ca	P	Mg
16.80	11.61	1.67	0.79

Fig. SM4. SEM image (a), spatial distribution of the most abundant elements represented by Ca (b), P(c) and Mg (d) and EDX analysis (e) on a cross sections of the two special Malpighian tubules ("lime glands") of the young larvae of 2-3 mm (supposed to belong to the second/third instar) of *H. illucens*. Typical EDX spectrum on the granules **(e)** and on the cuticular surface (f) shows the presence of a high amount of Ca and traces of P and Mg (atomic percentage) in the granules filling the Malpighian tubules.
